# Adsorption of the ionic liquid [BMP][TFSA] on Au(111) and Ag(111): substrate effects on the structure formation investigated by STM

**DOI:** 10.3762/bjnano.4.102

**Published:** 2013-12-16

**Authors:** Benedikt Uhl, Florian Buchner, Dorothea Alwast, Nadja Wagner, R Jürgen Behm

**Affiliations:** 1 Institute of Surface Chemistry and Catalysis, University Ulm, Albert-Einstein-Allee 47, D-89081 Ulm, Germany; 2 Helmholtz Institute Ulm Electrochemical Energy Storage (HIU), Albert-Einstein-Allee 11, D-89081 Ulm, Germany

**Keywords:** adsorption, Ag, Au, [BMP][TFSA], ionic liquids, scanning tunnelling microscopy, self-assembly

## Abstract

In order to resolve substrate effects on the adlayer structure and structure formation and on the substrate–adsorbate and adsorbate–adsorbate interactions, we investigated the adsorption of thin films of the ionic liquid (IL) 1-butyl-1-methylpyrrolidinium-bis(trifluoromethylsulfonyl)imide [BMP][TFSA] on the close-packed Ag(111) and Au(111) surfaces by scanning tunneling microscopy, under ultra high vacuum (UHV) conditions in the temperature range between about 100 K and 293 K. At room temperature, highly mobile 2D liquid adsorbate phases were observed on both surfaces. At low temperatures, around 100 K, different adsorbed IL phases were found to coexist on these surfaces, both on silver and gold: a long-range ordered (‘2D crystalline’) phase and a short-range ordered (‘2D glass’) phase. Both phases exhibit different characteristics on the two surfaces. On Au(111), the surface reconstruction plays a major role in the structure formation of the 2D crystalline phase. In combination with recent density functional theory calculations, the sub-molecularly resolved STM images allow to clearly discriminate between the [BMP]^+^ cation and [TFSA]^−^ anion.

## Introduction

In the last 15 years ionic liquids (ILs) have attracted increasing attention due to their special physical and chemical properties such as a low volatility, high chemical stability, low flammability, high intrinsic conductivity, high polarity, nearly vanishing vapour pressure and their wide electrochemical window [1–3]. Because of the enormous flexibility in varying the combination and nature of cations and anions [4], e.g., by using different alkyl chain lengths at the cations [2,5–7] it is possible to systematically optimize ionic liquids for a specific application. Aside from many other applications, ionic liquids have been proposed as promising new solvents in electrochemical applications, e.g., in lithium ion batteries [8–10]. For the latter application, trifluoromethylsulfonyl imide [TFSA] based ionic liquids have turned out to be promising candidates; members of this group, e.g., alkylmethylpyrrolidinium-[TFSA] seem to suppress dendrite formation [11]. The underlying molecular processes, however, are not yet understood. Thus, a systematic and fundamental understanding of the interface between ionic liquids and the respective electrode surface (solid–liquid interface) is essential for developing improved future battery systems based on ILs. Correspondingly, the interaction between different ILs and various electrode materials was investigated by electrochemical methods, including, e.g., cyclovoltammetry, but also by other techniques such as in situ scanning tunnelling microscopy [12–14].

More detailed insight, on a molecular scale, may be gained in model studies investigating the interface between the respective solid surface and thin films of the IL under ultrahigh vacuum (UHV) conditions. These films can be deposited by physical vapour deposition, which allows to accurately control the film thickness (coverage) in the submono- to multilayer regime. Furthermore, applying proper cleaning procedures, high purity films can be obtained. This not only allows to use a wide variety of surface science tools for characterization of the IL adsorbates/adlayers, but also to vary the temperature over a wide range, down to cryogenic temperatures, where molecular motion is largely frozen. This way, the interaction between substrate and adsorbed ILs was investigated in a number of studies, applying both spectroscopic techniques such as ultraviolet photoelectron spectroscopy (UPS) [15–16], X-ray photoelectron spectroscopy (XPS) [17–21], or temperature programmed desorption (TPD) [22], as well as scanning probe microscopies (scanning tunnelling microscopy (STM) and atomic force microscopy (AFM)) [16,23–24]. These surface science techniques allow to gain detailed information on the electronic properties of the ILs and adsorption induced modifications therein, on the chemical nature of the adsorbed species, and on the structure and structure formation in the resulting adlayer. The latter in turn provides information on the molecule–substrate and molecule–molecule interactions in the respective adsorption system.

**Figure 1:**
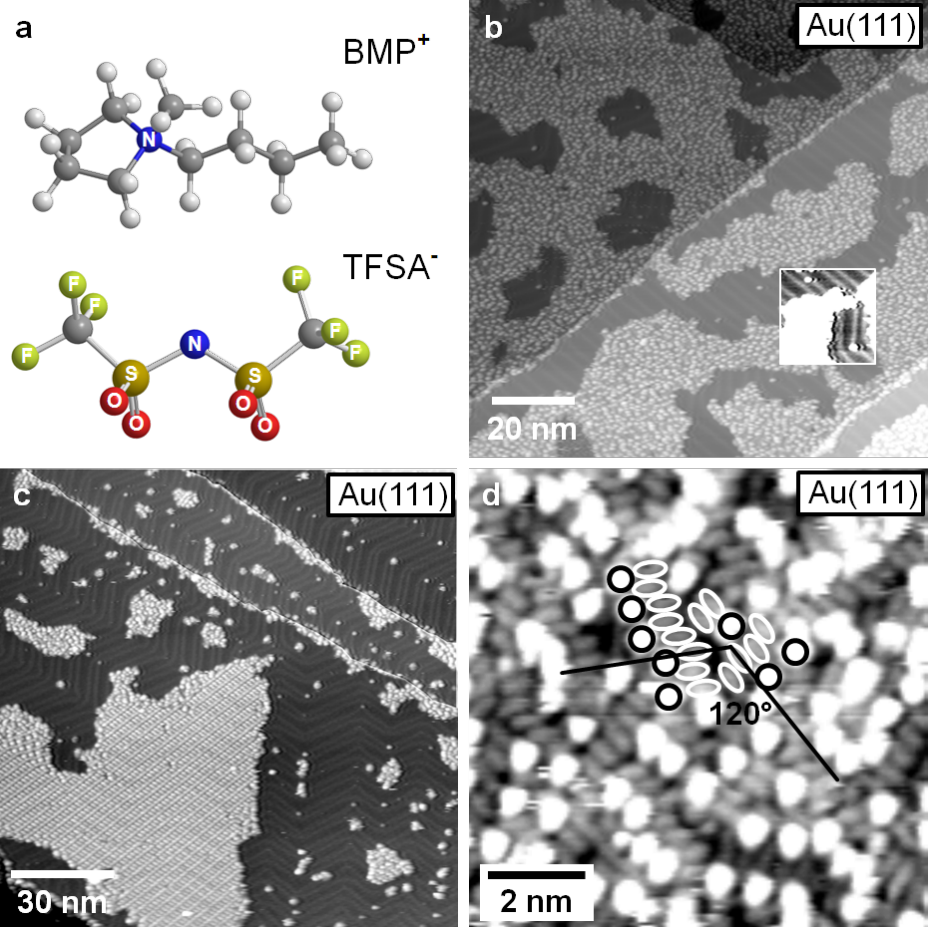
(a) Ball and stick model of 1-butyl-1-methylpyrrolidinium-bis(trifluoromethylsulfonyl)imide [BMP][TFSA] (grey: C, white: H, blue: N, red: O, green: S, yellow: F) (drawn with Chem3D). (b) STM image of a Au(111) surface covered with 0.7 ML of [BMP][TFSA] arranged in islands of the 2D glass phase. Inset: detail of the image in (b) with enhanced contrast between the adsorbate islands, resolving the Au(111) reconstruction pattern (*T* = 112 K, *U*_T_ = −1.9 V, *I*_T_ = −40 pA); (c) STM image of a Au(111) surface with a small amount (≈0.2 ML) of adsorbed [BMP][TFSA], resolving the preferential decoration of steps and the nucleation of small islands with 2D glass structure at the elbows of the Au(111) reconstruction, while islands with a 2D crystalline structure have grown larger. The Au(111) reconstruction pattern is visible on the uncovered parts of the surface (*T* = 111 K, *U*_T_ = −1.74 V, *I*_T_ = −0.020 nA). (d) High resolution image of the 2D glass phase on Au(111): longish protrusions with a lower height are visible between the round shaped protrusions (partly marked by white circles and ellipsoids) (*T* = 119 K, *U*_T_ = −1.06 V, *I*_T_ = 80 pA).

In the following, we will discuss these aspects for the adsorption of 1-butyl-1-methylpyrrolidinium-bis(trifluoromethylsulfonyl)imide [BMP][TFSA] (ball and stick models of the ions are shown in Figure 1a) comparing adsorption on the close-packed surfaces of Au and Ag. In that comparison, we will make use of new and recently published data [25–26]. In addition to their different chemical nature, these surfaces differ from each other in that the Au(111) surface is reconstructed, forming the well-known herringbone reconstruction [27], while the Ag(111) surface is not reconstructed. We will focus on questions related to structure and structure formation such as the nucleation and growth behavior and temperature effects thereon, the nature and stability of ordered phases, or the role of the substrate. First we will discuss the adsorption behavior for room temperature adsorption, then concentrate on the structure formation at low temperatures down to 100 K, and finally elucidate the thermal stability of the different adlayer phases.

## Results and Discussion

### Room temperature adsorption

Previous STM studies by Waldmann et al. and by Foulston et al. on the structure and structure formation of IL thin films on single crystal substrates, specifically for 1-butyl-1-methylpyrrolidinium-tris(pentafluoroethyl)trifluorophosphate [BMP][FAP] adsorption on Au(111) [24] and for 1-ethyl-3-methylimidazolium-[TFSA] ([EMIM][TFSA]) adsorption on Au(110) [16], respectively, indicated that at room temperature the thermal mobility of IL adsorbates is too high for resolving individual molecular entities by STM. Images recorded under these conditions resolved a characteristic noise in the tunnel current on the IL covered surfaces, which was not observed in the absence of the IL adlayer. The authors of those studies attributed this noise to the formation of a 2D gas or 2D liquid adlayer phase, where the IL adsorbates are mobile on the surface and cause a temporary modification in the tunnel current whenever a diffusing admolecule passes through the tunnel gap underneath the tip. (Note that the 2D gas and 2D liquid adlayer phase differ mainly by the adlayer density.) Similar effects were observed also for adsorption of [BMP][TFSA] on Au(111) [25] and on Ag(111) [26]. While this point shall be discussed in more detail later, it should be noted here already that the high mobility of the adsorbed species, which reflects a low lateral corrugation of the adsorption potential along the surface, is incompatible with the formation of localized covalent bonds between substrate and the adsorbed IL species.

Finally it should be noted that the STM images showed no indications of a restructuring of the Ag(111) or Au(111) surfaces upon interaction with [BMP][TFSA], as it was reported by Atkin et al. [23] for [BMP][TFSA] on Au(111) in electrochemical STM measurements, where bulk IL was in contact with the surface at potentials between −0.4 and −2.2 V vs the ferrocene/ferrocenium (Fc/Fc^+^) redox couple [28]. Hence, the presence of the IL adsorbate alone is not sufficient to induce a restructuring of the substrate surface.

The information derived from STM imaging can be combined with results of spectroscopic measurements. XP spectra presented in [25] for submono- to multilayer [BMP][TFSA] films on Au(111) showed a similar dependence of the intensity of the different XPS signals (C(1s) and N(1s)) on the emission angle and an XPS based composition identical to the stoichiometric ratio, both in the submono- to monolayer regime and at higher coverages. Therefore, the authors of that study concluded that in average all atoms of the two ions are located in the same layer, with anions and cations placed side by side on the surface. Therefore, both ions in the first layer are in direct contact with the surface. This is also confirmed by the fact that for coverages up to 1 monolayer (ML) the C(1s) and N(1s) XPS signals show a shift of 1.1 eV to lower binding energy (BE), due to the interaction with the Au(111) surface. For Ag(111), where ARXPS measurements are not available, we expect a comparable adsorption behavior. This is supported also by the results of density functional theory (DFT) calculations discussed below.

These results can be compared with findings reported for other IL adsorption systems. For 1,3-dimethylimidazolium-[TFSA] ([MMIM][TFSA]) and 1-octyl-3-methylimidazolium-[TFSA] ([OMIM][TFSA]) adsorption on Au(111) [19], the same adsorption geometry with both the anion and cation in direct contact to the surface was concluded from ARXPS measurements at room temperature. The authors of that study deduced that the cation adsorbs with the imidazolium ring flat on the surface and that the anion adsorbs in a cis-conformation, with the SO_2_-groups pointing to the surface and the CF_3_-groups pointing towards the vacuum. The same adsorption geometry for the anion was also proposed by Sobota et al. [29] for [BMIM][TFSA] (B = butyl) adsorbed on a thin alumina film grown on NiAl(110) [30–31], utilizing a combination of infrared reflection absorption spectroscopy (IRAS) and density functional theory (DFT) calculations. [OMIM][TFSA], which differs from [MMIM][TFSA] only by its longer alkyl chain, showed a coverage dependent adsorption geometry on Au(111): at coverages below 0.6 ML, the octyl chain lies flat on the surface, while at higher coverages it sticks up from the surface, reducing the space requirement of the adsorbed ion pair. In contrast, for adsorption on other surfaces, also other adsorption geometries were reported: For [EMIM][TFSA] adsorption on a glass substrate, an adsorption geometry with the cations lying flat in direct contact with the surface and the anions placed on top of the cations was proposed based on ARXPS measurements [17]. For [MMIM][TFSA] adsorption on Ni(111) [20], a similar adsorption geometry was proposed for adlayers in the submonolayer coverage regime up to ≈0.8 ML. Finally, for coverages >0.8 ML, the ARXPS data did not show a vertical layering of the different ions, therefore under these conditions both adsorbed cations and anions have to be in direct contact to the substrate. This behaviour was explained by an increasing repulsive electrostatic interaction between the ion pairs with increasing coverage, leaving the former configuration energetically less favourable at coverages above 0.8 ML compared to a structure with both species in direct contact with the surface.

Overall, though structural resolution of the IL adlayer was not possible at room temperature, the examples discussed above, with their very similar ILs (most of them contain the same anion and an imidazolium- or pyrrolidinium-based cation), demonstrate already that the structures resulting in ionic liquid adlayers depend sensitively on the substrate. This will become even more evident when comparing adlayer structures on the two different surfaces Ag(111) and Au(111) in the next section.

### Low-temperature adsorption

The situation changes considerably when cooling the samples to lower temperatures. Under these conditions, molecular motion is frozen and the adsorbates can be resolved in STM measurements. Since cool-down was done very slowly (ca. 2 K min^−1^), the system stays in thermal equilibrium until the adsorbates are immobilized and STM images show the surface at this freezing temperature. Although the resulting adlayer differs clearly from that in the solid–liquid interface at room temperature and above, e.g., by the much higher molecular mobility, these measurements provide sensitive information on the interactions between the adsorbed ions and on the variation in substrate–adsorbate interaction (adsorption potential) along the surface. These characteristic energies can be used as starting point also for the description of the solid–liquid interface at room temperature and above.

In their STM study on [BMP][FAP] adsorption on Au(111), Waldmann et al. resolved round shaped protrusions at temperatures below 210 K [24]. A direct assignment of these structures to adsorbed cations or anions and a clear identification of the adlayer structure in terms of co-planar adsorption of both types of ions or adsorption of one species on top of the other one, however, was not possible from these data. Likewise, in their STM study of [EMIM][TFSA] adsorption on Au(110), Foulston et al. identified round shaped protrusions at liquid nitrogen temperature, which were oriented along the missing row lines of the (1 × 2) reconstruction of the Au(110) surface, but without long-range ordering along the lines or strict correlations between neighbouring lines. These protrusions were proposed to represent the complete IL ion pair. Also in these images it was not possible to resolve and identify anions and cations [16]. Overall, these studies succeeded in resolving individual molecular entities, but were not able to derive the actual structure of the adlayer, or to identify anions and cations separately.

Going to the present system, [BMP][TFSA] adsorption on Ag(111) and Au(111), we find two types of structures, one type which similar to the above observations does not exhibit a long-range order but rather a short-range order, which we denote as ’2D glass’ phase, and a second one exhibiting a distinct long-range order [25–26]. This latter structure can be denoted as ‘2D crystalline’ phase.

**Figure 2:**
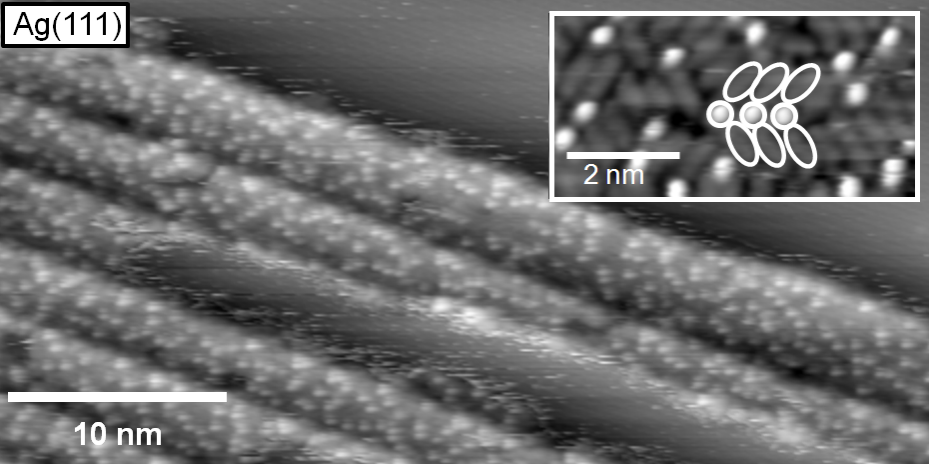
STM image of a submonolayer film of [BMP][TFSA] adsorbed on Ag(111); the narrow terraces of the surface are covered with IL islands in the 2D glass phase, the inset shows a high resolution image of the 2D glass structure resolving both the round shaped and the longish protrusions (marked with white circles and ellipsoids) (*T* = 135 K, *U*_T_ = −1.14 mV, *I*_T_ = 100 pA).

Examples for the ‘2D glass’ adlayer structure are shown in Figure 1 for adsorption on Au(111) and later in Figure 2 for adsorption on Ag(111). STM images of the 2D crystalline structures are depicted in Figures 4–6 (see below).

We will first concentrate on the discussion of the ‘2D glass’ structure. In Figure 1b, a Au(111) surface covered with 0.7 monolayers (ML) of [BMP][TFSA] adsorbates is shown (for a definition of 1 ML see Experimental section). In that image, the IL adsorbates appear as round shaped protrusions and form distinct islands on the surface. In between the islands, adsorbate free Au(111) surface areas appear, where the typical [27] zig-zag pattern of the Au(111) surface reconstruction is resolved (see inset with enhanced contrast in Figure 1b). The formation of islands demonstrates the presence of attractive interactions between the adsorbed IL species, which must be strong enough to cause island formation at the freezing temperature. Interestingly, the steps of the Au(111) surface are decorated with adsorbate species, hence these sites seem to be preferred adsorption sides. While this is true for both the ascending and descending side of the steps, on the lower and upper terrace side, respectively, the structural characteristics differ for both sites. On the upper terrace side, a single row of IL adsorbates follows the step, indicative of a stronger adsorption at these sites, similar to the frequent observation of stronger adsorption of atomic adsorbates and adsorbed small molecules [32]. At the lower terrace side, the IL adsorbates seem to condense at the ascending steps, forming large IL islands which grow over the Au(111) terraces. Interestingly, 2D condensation of IL adsorbates at the row of adsorbate species decorating the step edge on the upper terrace side is not possible. The physical reason for the different 2D condensation behavior on the upper and lower step edge is not yet clear.

In addition to the step edges, also the elbows of the Au(111) reconstruction act as nucleation sites for 2D island formation. A few examples are visible in Figure 1b. More clearly, this is observed in STM images recorded at low coverages, where only the steps and the elbows are covered with adsorbates, as illustrated in Figure 1c. This points to a higher adsorption energy at the elbow sites as compared to the other surface areas, similar to findings for metal epitaxy, e.g., Ni/Au(111) [33], or adsorption of large molecules such as porphyrin molecules [34].

The (short-range) ordering of the adsorbates in the islands was checked by calculating a Fourier transformation (FFT) in sections of STM images which show solely one island and the distribution of round shaped protrusions on it. The FFT always shows a broad circle (see [25]), as expected for a short-range ordered system. We found no evidence for a coverage effect on the density and structural characteristics of this phase in the submonolayer and monolayer regime.

On Ag(111), adsorption of [BMP][TFSA] leads to a similar ‘2D glass’ structure. In this case, however, it is formed only on narrow terraces with a width of ≤10 nm, as can be seen exemplarily in the STM image in Figure 2, while on Au(111) there was no obvious influence of the terrace width discernible.

This difference is most easily explained by the presence/absence of the Au(111) reconstruction pattern, which seems to severely affect the ordering behavior. Keeping in mind that on Au(111) the elbows of the surface reconstruction act as nucleation sites for IL island formation, the larger tendency for disordered 2D structures on Au(111) can at least partly be ascribed to a mismatch between the lattice created by the elbow sites and the ordered lattices of IL adsorbates (see below). In that case, IL adsorbate islands created at neighbouring elbow sites are not in registry, and therefore can not coalesce easily. These effects are absent on the unreconstructed Ag(111) surface.

In the inset of Figure 2, we show a high resolution image of the 2D glass structure. It is recorded in the central area of an island with very little or no motion of the adsorbed molecules during imaging. Between the round shaped protrusions, longish protrusions with a lower apparent height are resolved. Some of these species are marked in the image by white circles and ovals for better identification. For Au(111), high resolution images of the disordered structure look exactly the same, with identical structures, mean distances between the protrusions etc. (see Figure 1d and inset in Figure 2). Therefore, the adsorption geometry, the structure formation and the molecule–molecule and molecule–substrate interactions in the 2D glass structure of [BMP][TFSA] should be identical on Au(111) and Ag(111) and they can be discussed for both substrates together. The first question relates to the origin of the different protrusions in the STM images. Most simply, the longish protrusions represent one ion type and the round shaped protrusions the other one. In that case, the adsorbed cations as well as the anions lie next to each other in direct contact to the surface, as it was already concluded from the XPS data for [BMP][TFSA] on Au(111) [25] and for the adsorption of the very similar ILs [MMIM][TFSA] and [OMIM][TFSA] on Au(111) [19]. A quantitative evaluation of the numbers of longish and round shaped protrusions in several 2D glass domains and on several STM images yielded a ratio of 2:1. This leaves us with two different plausible explanations: either one ion type is represented by two parallel longish protrusions and the other one by the round shaped protrusion, or one type is represented by the round shaped protrusion plus one longish protrusion and the other one by the other longish protrusion. Though the first interpretation sounds more convincing, this question cannot be solved on the basis of the STM images alone. We will get back to this point after discussion of the 2D crystalline structure.

As evident in Figure 1d, the longish protrusions are aligned in rows of varying lengths (between 2 and 8 protrusions are typical), which are oriented at an angle of roughly 120° (or 240°) in between. The resulting threefold symmetry is probably due to an alignment to the closed packed directions of the Au(111) surface. So the structure is not completely random, even if there is no long-range order visible for the distribution of the round shaped protrusions in the FFT.

**Figure 3:**
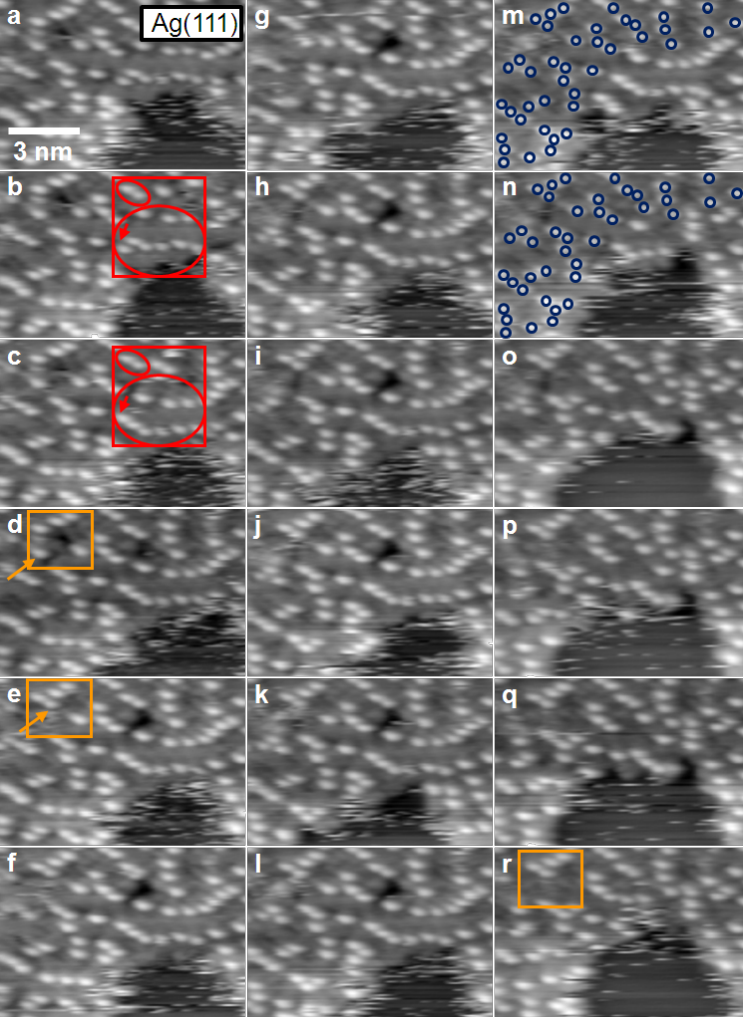
Sequence of STM images of [BMP][TFSA] adsorbed on Ag(111), acquired at 124 K, imaging the phase boundary between the 2D glass and 2D liquid phase (image-to-image time ≈11 s). Noisy features near the phase boundary and the successively changing phase boundary are indications for mobility at the phase boundary. A red frame in Figure 3b and 3c including two ovals marks two protrusions at stable positions (smaller oval), while the other protrusions in the larger oval shift to a lower position. The red arrow points out the changing position of a single protrusion. The orange boxes in Figure 3d and 3e highlight a molecular jump between two consecutive images. Subsequently, no motion is observed up to Figure 3r. The blue circles in Figure 3m and 3n show stable protrusions, while others at and close to the boundary between 2D glass structure and adsorbate free area clearly change positions (*T* = 124 K, *U*_T_ = −0.76 V mV, *I*_T_ = 50 pA).

In addition to the differences in the structure formation processes between Au(111) and Ag(111), there seem to be differences also in the mobility of the IL adsorbates on these two surfaces. For the Ag(111) surface, apparently adsorbate free areas between IL adsorbate islands, e.g., on the central terrace in Figure 2 or in front of the topmost step in this image, show a significant noise. The noisy appearance resembles that obtained for imaging at room temperature, but in the latter case the noise is more pronounced and present on the entire terrace. On Au(111), this noise is visible also on similarly covered surfaces for STM imaging at 100 K, but is much less pronounced. This indicates that these areas are essentially free of mobile IL adsorbates. A higher mobility of IL adsorbates on Ag(111) compared to Au(111) is evident also from inspection of series of images from the same surface area, which reveal changes in the island boundaries with time. This is illustrated in Figure 3, which shows a time sequence of STM images (time between subsequent image starts: 11 s) recorded on a partly IL adsorbate covered Ag(111) surface. Beside the 2D glass phase, an apparently uncovered region is visible, which, as also described for the STM image in Figure 2, appears noisy. This sequence clearly demonstrates that the island edge (phase boundary) gradually changes with time (Figure 3a–r). While the major part of the round shaped protrusions persists on the same site from frame to frame, molecular jumps are detected for others. This is evident, e.g., in the areas marked with red ovals in Figure 3b and 3c. The two protrusions in the smaller oval are stable from image to image, while the protrusions in the larger oval collectively move to a lower position in the image. A red arrow is also included, pointing towards a single protrusion, which changed position. In Figure 3d and 3e, the arrows in the orange frame mark a molecular jump between two consecutive images, while in subsequent images no motion at the same position takes place (Figure 3r). In Figure 3m and 3n, the blue circles label protrusions, which persist at the same positions, while for others at and close to the boundary between 2D glass structure and adsorbate free area significant changes are visible. Thus, both temporary changes directly at the phase boundary and also some limited motion inside the 2D glass phase is found on Ag(111). On Au(111), these processes were also observed, but less frequent. These structural changes can be explained either by a motion of IL adsorbates along the island edge or by a 2D adsorption–desorption equilibrium between the IL adsorbate islands and a 2D gas/liquid of IL adsorbates. As expected for this case, structural variations mainly take place at the island perimeter, while the inner part of the islands is essentially stable; with infrequent molecular jumps only in the vicinity of defects in the adlayer lattice.

**Figure 4:**
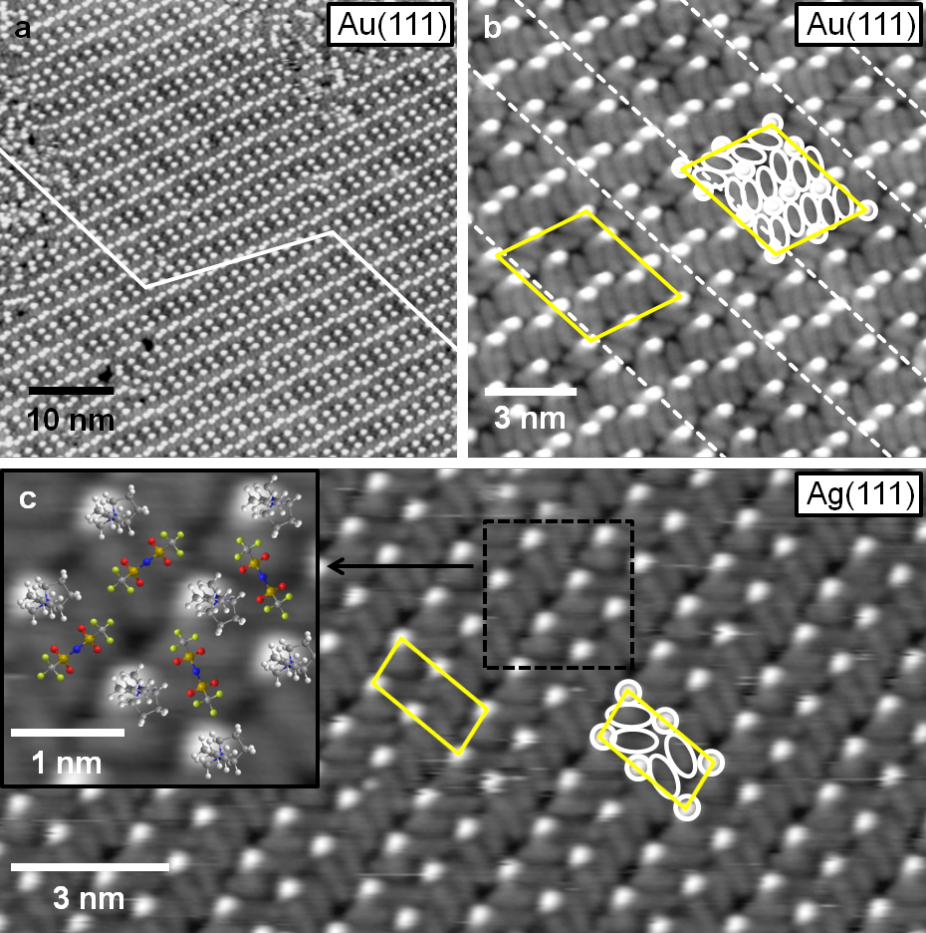
High resolution STM images of the 2D crystalline structures on Au(111) (a, b) and Ag(111) (c). The unit cells are marked with yellow lines. Both structures are composed from round shaped and longish protrusions, which are marked with white circles and ellipsoids. In (a) the white line and in (b) the dashed lines mark the zig-zag lines of the Au(111) reconstruction, which are visible through the 2D crystalline structure of the IL adsorbates; the inset of (c) shows an enlarged part of image (c) with superimposed ball and stick models of [BMP][TFSA] (a: *T* = 139 K, *U*_T_ = −1.20 V, *I*_T_ = −0.060 nA; b: *T* = 116 K, *U*_T_ = −0.71 V, *I*_T_ = −0.10 nA; c: *T* = 134 K, *U*_T_ = −0.37 mV, *I*_T_ = 110 pA).

Aside the 2D glass phase, also well ordered, 2D crystalline domains/islands are found on the surface. This is illustrated in the high resolution images of the 2D crystalline structure on Au(111) in Figure 4a and 4b. These images also reveal characteristic round shaped protrusions and in between longish, less pronounced protrusions. Similar to the findings in the 2D glass phase, the ratio between round and longish protrusions is 1:2. In the one lattice direction, the round shaped protrusions form a densely packed line of dimers, which are slightly rotated against the main direction of the line (in Figure 4a, the lines run roughly from the lower left to the upper right corner), which results in a zig-zag like appearance. Between two close-packed lines of round protrusions, there are always parallel lines with a lower density of these protrusions (50%). The resulting unit cell is marked yellow in Figure 4b. The longish protrusions are also aligned in row like structures, which run in the same direction as those formed by the round shaped protrusions (see second unit cell marked in Figure 4b, where the round and longish protrusions are marked by ovals and circles). Also in this case, there are two types of rows. In two neighboured rows the longish protrusions are oriented in the same direction. In the subsequent third row, they are rotated by ≈120°. In the latter row, the density of longish protrusions is only two thirds of that in the other two rows (4 instead of 6 longish protrusions per row and unit cell). The size of the unit cell seems to differ slightly, depending on whether the 2D crystalline domain is completely surrounded by a 2D glass domain, i.e., whether the surface is saturated with a monolayer of IL adsorbate, or whether there are adsorbate free surface areas around (= submonolayer coverage regime). The ordered domains in Figure 4a and 4b were recorded on a surface covered by a submonolayer film; in this case the unit cell has a size of 4.20 ± 0.04 nm × 3.37 ± 0.04 nm with an angle of 68 ± 2° in between. In the monolayer coverage regime, the dimension of the unit cell shrunk to 3.79 ± 0.04 nm × 2.89 ± 0.04 nm, with an angle of 78 ± 2° in between, indicative of a certain flexibility in the structural arrangement of the adlayer. In both cases, the unit cell contains 8 round shaped and 16 longish protrusions, which most likely (see below) corresponds to 8 ion pairs of adsorbed [BMP][TFSA]. This gives a space requirement for one ion pair of 1.64 nm^2^ in the submonolayer and 1.34 nm^2^ in the monolayer coverage regime, equivalent to densities of 0.61 and 0.75 ion pairs per nm^2^, respectively (see Table 1). The alignment of the unit cell with respect to the substrate lattice will be discussed below.

**Table 1:** Summary of the adsorbate densities and melting temperatures of the adlayer phases found on Ag(111) and Au(111).

IL adsorbate phase	density / nm^−2^	2D melting temperature / K^−1^

2D crystalline phase / Au(111), submonolayer coverage regime	0.61 ± 0.03	170 ± 5
2D crystalline phase / Au(111), monolayer coverage regime	0.75 ± 0.03	225 ± 5
2D glass phase / Au(111), submonolayer coverage regime	0.61 ± 0.03	113 ± 5
2D glass phase / Au(111), monolayer coverage regime	0.61 ± 0.03	173 ± 5
2D crystalline phase / Ag(111), submonolayer coverage regime	0.79 ± 0.03	180 ± 10
2D crystalline phase / Ag(111), monolayer coverage regime	0.79 ± 0.03	180 ± 10

Atkin et al. [23] concluded from their AFM measurements that the first [BMP][TFSA] adlayer binds more strongly than the following layers, i.e., it binds more strongly to the metallic substrate than to itself. In that case, one may expect the saturation density in the first layer to be higher than in the bulk phase. For the present adsorption system this means that the bulk structure may be more similar to the ordered phase in the submonolayer coverage regime than to that at monolayer saturation.

The 2D crystalline structure of [BMP][TFSA] on Ag(111), shown in Figure 4c, is more simple than the one formed on Au(111). The round shaped protrusion are aligned in rows, running from the bottom left side to the top right side in Figure 4c. The spacing between these rows is slightly different, leading to the appearance of pairs of lines. In between these lines, the longish protrusions are also aligned in the same direction. The orientation of the long side of these protrusion changes by 120° between neighbouring rows (in the limits of the experimental accuracy), i.e, they are parallel to each other in every second row. In the row of longish protrusions that lies between the two more widely spaced rows of round shaped protrusion, the longish protrusions are aligned in a straight line (parallel to the row of round protrusions), in the neighbouring lines the longish protrusions are pairwise rotated away from the direction of the row, which allows a closer spacing between the neighbouring rows of round shaped protrusions. The unit cell of this structure is marked twice in Figure 4c with yellow lines; in one of these cases, the protrusions in the unit cell are marked by white circles and ovals. The size of the unit cell is 1.1 ± 0.1 nm × 2.3 ± 0.1 nm with an angle of 95 ± 3° in between the two lattice directions. For Ag(111), the size (2.5 nm^2^) and geometry of the unit cell was found to be independent of the IL adsorbate coverage. The unit cell contains 2 round and 4 longish protrusions, which represent two [BMP][TFSA] ion pairs (see below). In that case, the space requirement per IL ion pair is 1.25 nm^2^, the density of the adsorbed ion pairs is 0.79 nm^−2^. This is very similar to the density of ion pairs on Au(111) in the monolayer regime, while in the submonolayer regime the ion pairs on Au(111) have a 30% lower density.

**Figure 5:**
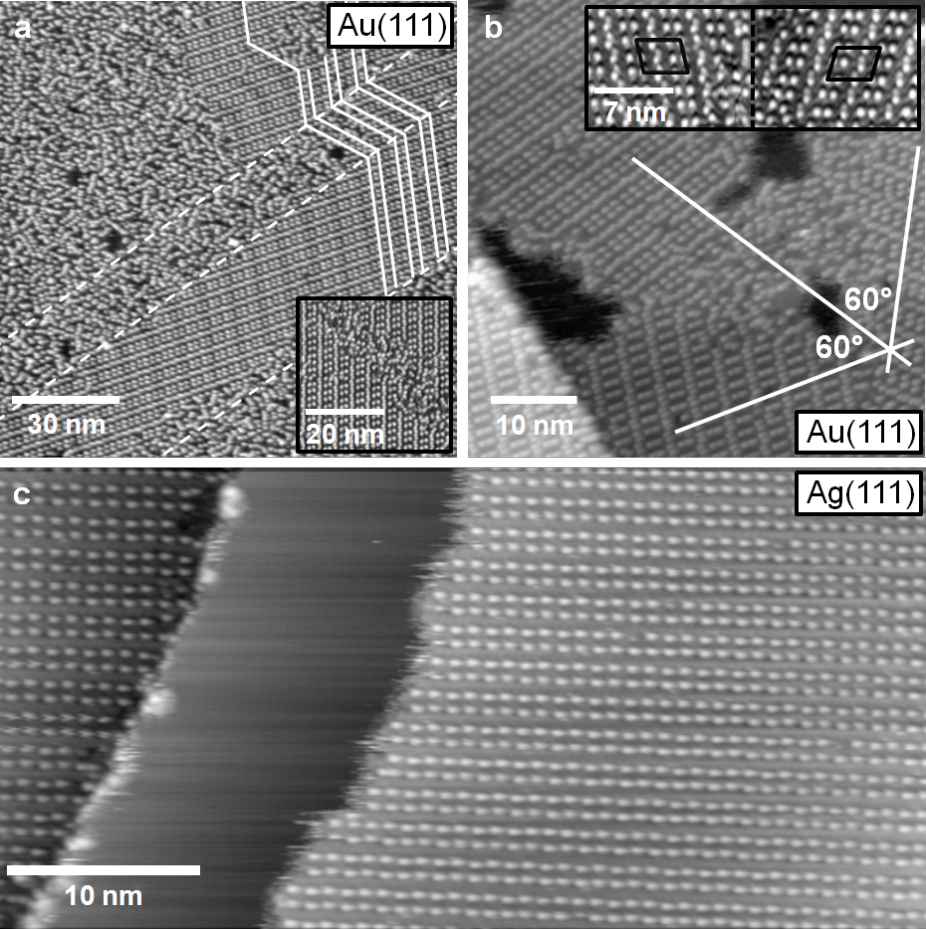
(a) STM image of a monolayer film of [BMP][TFSA] on Au(111), showing both 2D crystalline islands as well as 2D glass areas. The Au(111) reconstruction is visible in the 2D crystalline island (for better visibility it is marked with white lines in the upper right of the image). In the 2D glass domain, this is not resolved; dashed lines mark the domain boundaries of the Au(111) reconstruction pattern (*T* = 118 K, *U*_T_ = −1.25 V, *I*_T_ = −0.060 nA). (b) STM image of 2D crystalline domains of [BMP][TFSA] on a single Au(111) terrace in direct contact to each other. The domains are rotated by 60° to each other (*T* = 146 K, *U*_T_ = −1.20 V, *I*_T_ = 60 pA). (c) STM image of a submonolayer film of [BMP][TFSA] on Ag(111). The adlayer islands nearly completely consist of the 2D crystalline structure. The island boundary shows a frizzy appearance, which is associated with mobility of the adsorbed IL species, either along the island edge or in a 2D adsorption–desorption equilibrium between the 2D solid and the adjacent 2D gas phase (*T* = 130 K, *U*_T_ = −1.09 V, *I*_T_ = 80 pA).

Next we will discuss additional aspects of the 2D crystalline phase, such as its alignment with respect to the substrate surface lattice, its distribution structure on the surface, etc. The orientation of the IL adlayer can be derived from larger scale images as shown in Figure 5a and 5b for Au(111). In the image in Figure 5a, the Au(111) surface was covered with 1 ML of [BMP][TFSA]. The image shows one island of the 2D crystalline structure, which is surrounded by the 2D glass structure, as typical for the monolayer regime. The amount of the 2D crystalline structure relative to that of the 2D glassy was found to vary between experiments. In most cases, the amount of the 2D glass structure is higher than that of the 2D crystalline phase, and islands of the latter phase are embedded in a surrounding 2D glass phase. In the submonolayer regime (Figure 5b) this is similar, but the amount of 2D crystalline structure relative to that of the 2D glass phase is typically higher. This is illustrated in Figure 1c: on samples with a low coverage of [BMP][TFSA] adsorbates we only observed small islands with 2D glass structure (which are mostly growing from the elbow sites of the Au(111) reconstruction pattern), while the islands of the 2D crystalline structure present in between are much larger. The physical reason for the higher fraction of 2D crystalline phase at lower coverages, which reflects an easier alignment of the adsorbate species during cool-down under theses conditions, may only be speculated upon. It may be related to more stable adsorption at the perimeter of islands of the 2D crystalline phase compared to (small islands of) the 2D glass phase, which allows preferential growth of the former ones during cool down at lower coverages, while at higher coverage such effects do not seem to play a significant role.

The STM image in Figure 5a reveals another phenomenon typical for [BMP][TFSA] on Au(111). The 2D crystalline structure is also severely affected by the reconstruction pattern of the Au(111) surface. In this image, the zig-zag line pairs of the herringbone reconstruction are clearly visible through the adlayer, they are marked in Figure 5a with white lines in the upper right part to guide the eye. Note that for the 2D glass phase the reconstruction could not be resolved. In Figure 4a and 4b, the reconstruction pattern is also visible, but less pronounced. It is marked by a white line in Figure 4a. The adlayer is usually aligned in such a way that the direction of the longer side of the unit cell (see Figure 4b) is parallel to the lines of the Au(111) reconstruction pattern. Accordingly, the 2D crystalline phase tends to grow in domains/islands which are limited by the domain boundaries of the herringbone reconstruction, i.e., by the bending points of the dislocation lines. This can be seen in Figure 5a, where the positions of the bending points of the Au(111) surface reconstruction are connected with white dashed lines. A large part of the 2D crystalline domain visible on this image, which extends diagonally across the image, grows on one domain of the Au(111) reconstruction and fills it nearly completely. As can be seen in the upper part of the image, it is also possible for the adlayer structure to grow across such kind of domain boundary in the Au(111) reconstruction pattern. This was only observed, however, when the adlayer domain spanned at least over three Au(111) reconstruction domains and the part with the ‘wrong’ orientation is in the middle. In this case we often observed a narrow stripe of 2D glass phase directly at the elbows of the Au(111) reconstruction pattern (see inset in Figure 5a). Isolated 2D crystalline islands, which are limited to a single domain of the Au(111) reconstruction and where the rotational orientation of the adlayer island, as described above, does not fit to the orientation of the Au(111) reconstruction, have not been observed. It is interesting to note that the elbows of the Au(111) reconstruction act as nucleation sites for nucleation of 2D glass phase islands, and on the other hand limit domains of the 2D crystalline phase, which seems to be in contrast to each other. A simple physical explanation is still missing.

Because of the threefold symmetry of the Au(111) surface and of the Au(111) reconstruction pattern there are only three different orientations for the 2D crystalline domain on the surface possible. In Figure 5b, three 2D crystalline domains are present which are rotated at angles of 120° relative to each other. Furthermore, because of the non-rectangular form of the adlayer unit cell, two different chiral forms of that unit cell (see Figure 4b) are possible along each direction, leading to 6 possible adlayer domains in total. An example for two islands with chiral structure is shown in the inset in Figure 5b.

On Ag(111), the situation is very different because of the absence of a surface reconstruction. In this case the domains of the 2D crystalline structure mostly extend across the entire terraces, i.e., the domains extend across hundreds of nanometers (if the surface is well prepared and the terraces are sufficiently large). This is equally true also for islands of the 2D crystalline phase in the submonolayer coverage regime, where these islands coexist with large areas of adsorbate free surface. At typical images sizes, most of the STM images show either a fully covered or an adsorbate free surface. Small terraces with a width ≤10 nm are covered with ILs adsorbed in the 2D glass structure as described above. The 2D crystalline structure is normally attached to an ascending Ag(111) step, mostly with a small amount of the 2D glass structure between step and ordered adlayer phase. In this case, the width of the 2D glass phase is between a few molecules to several nm. It seems as if the steps of the substrate surface disturb the formation of the 2D crystalline structure, rather than acting as nucleation sites. When comparing different domains (an example is shown in Figure 5c) of the 2D crystalline structure, they are all aligned in the same direction to each other (like in Figure 5c) or at angles of 60° or 120° to each other, even when they grow on different terraces of the substrate. This suggests that the adlayer structure also follows the threefold geometry of the Ag(111) surface. Due to experimental reasons (adlayer imaging requires a large tunnel resistance while atomic resolution require low tunnel resistances) it was not possible to achieve atomic resolution of the surface near a boundary of a 2D crystalline island, therefore it was not possible to correlate the adlayer orientation directly with the substrate lattice.

In addition to the different arrangements of [BMP][TFSA] on Au(111) and Ag(111), we also found differences in the mobility of the island edges of the 2D crystalline phase, evidenced by a frizzy appearance of the island edges (Figure 5b and 5c). The frizzyness of the island boundary is proportional to the displacement of the boundary position between subsequent images, which arises from 2D adsorption/desorption of molecules at the island perimeter or diffusion of adsorbates along the island perimeter. The displacement can be quantified by determining the change in position of the island boundary in successive STM line scans. A quantitative evaluation revealed that the root mean square deviation of the position is more than double for Ag(111) (see Figure 5c) than for Au(111) (Figure 5b), indicative of a significantly higher mobility of the adsorbates at the island perimeters on the Ag(111) surface than on Au(111).

**Figure 6:**
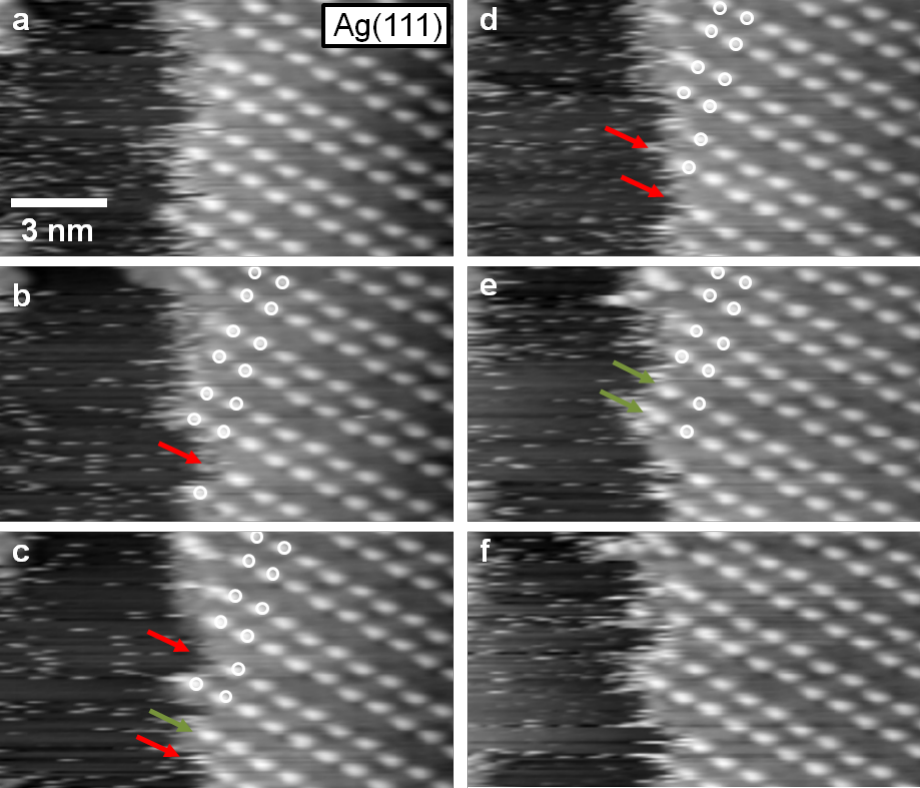
Time sequence of STM images at the phase boundary of the 2D crystalline phase of [BMP][TFSA] on Ag(111), recorded at 124 K (image to image time of ≈11 s) The images exhibit frizzy features directly at the 2D crystalline | 2D liquid interface, reflecting mobility of the adsorbed IL species, either along the island edge or in a 2D adsorption–desorption equilibrium. The red arrows in the images show places at the boundary, where round shaped protrusions vanish compared to the preceding image. The green arrows depict locations where a protrusion is attached to the boundary (*T* = 124 K, *U*_T_ = −0.76 V, *I*_T_ = 50 pA).

The mobility of the IL adsorbates at the edge of a 2D crystalline adlayer island on Ag(111) is resolved in more detail in the sequence of STM images shown in Figure 6. The images were acquired at the same position with a frame to frame time of 11 s. It is clearly visible that the island edge changes with time. Places, where the round shaped protrusions vanished from one image to the other, are labelled with red arrows. Those places, where a round shaped protrusion is added to the structure are labelled with green arrows. Similarly as discussed for the mobility of the 2D glass phase on Ag(111), we assume that these changes are due to sudden motion of IL adsorbates along the island edge, or, more plausible, to 2D adsorption–desorption equilibrium between the IL adsorbate islands and a 2D gas/liquid of IL adsorbates. Again those regions, which are apparently free of adsorbate appear with streaky features, which we attribute to highly mobile molecules in a 2D gas/liquid phase, which diffuse to fast to be resolved with STM. Round shaped protrusion in the inner parts of the islands remain stable over time. The difference compared to the 2D glass phase, where infrequent jumps of these protrusions were possible, is explained by a higher stability and the absence of defects in the 2D crystalline phase.

Despite of the considerable structural insight gained from these STM images it was not possible to unambiguously identify the adsorbed IL species, specifically the adsorbed cations and anions in these images. This is possible by combination with dispersion corrected density functional theory (DFT-D) calculations, performed recently for adsorption of individual [BMP][TFSA] ion pairs on Ag(111) [26]. Details on the calculations can be found elsewhere [26].

According to those calculations, the ring of the cation lies flat on the surface and the butyl group points upwards. The anion adsorbs in a cis-configuration (both SO_2_-groups are positioned on the same side of the molecule, both CF_3_ groups on the other side, as it is also shown in Figure 1a) on the Ag(111) surface and binds via its two oxygen atoms to the surface. The CF_3_ groups point towards the vacuum. In this conformation both ion types of [BMP][TFSA] are in direct contact to the surface.

Simulated STM images using tunnelling conditions similar to the experimental ones (similar potential, comparable tip–surface separation) yield characteristic features very similar to those in the measured STM images. The upright standing butyl chain of the cation appears as round shaped protrusion. Right next to it an oval protrusion appears with lower height, which is due to the parts of the alkyl ring that are not directly lying below the butyl chain. In the measured STM images, only the round shaped protrusion is visible due to the limited resolution of the STM tip. The anion appears in the simulated images as two longish protrusions each of which is generated mainly by 2 fluorine atoms of the CF_3_-groups, in perfect agreement with experimental findings. Similar to experimental data, also their height is significantly lower than that of the round shaped protrusion reflecting the butyl chain of the cation.

Although these calculations did not include interactions between neighboured adsorbed ion pairs, the good agreement between the characteristic features in the experimental and calculated STM images are strong evidence for the validity of this assignment. Further support comes from the qualitative agreement with the ARXPS measurements of [BMP][TFSA], [MMIM][TFSA] and [OMIM][TFSA] adsorbed on Au(111) [19,25]. A suggestion for the structure model for BMP-TFSA on Ag(111) based on these data is shown in the inset of Figure 4c, where ball and stick models of the [BMP^+^] and [TFSA]^−^ ions are superimposed to the STM-image.

Another interesting result from these calculations was that based on a Bader charge analysis of the adsorption complex, the charges of the cation and the anion hardly change upon adsorption, and that the adsorption bond is dominated by van der Waals interactions. We expect these results as characteristic also for adsorption on Au(111).

### Thermal stability of the adlayer structure

Further information on adsorbate–adsorbate interactions can be derived from the thermal stability and the melting temperature of the structures on the surface. This was investigated by slowly heating up samples in the STM from 100 K to room temperature while recording STM images. Because of the very low heating rate (3 h for heating from 100 K to 300 K) the surface has enough time to maintain thermodynamic equilibrium during heating. Generally, the noise level in the STM images increased with rising temperature, implying a higher mobility of the 2D liquid on the surface. At certain temperatures it was finally not possible any more to resolve the adsorbate structures on the surface, which was interpreted as the temperature where the ion pairs, which before formed the island/domain started to move. This temperature is considered here as melting temperature (for the 2D glass structure it would be more correct to describe it as a glass transition when comparing to the notation in a bulk system, but for simplicity we use the term “melting temperature” for both adlayer structures). For the adlayer structures on the Au(111) surface, we could determine four different melting temperatures, which differ in a characteristic way: the 2D glass structure is stable up to a temperature of 113 ± 5 K in the submonolayer and up to 173 ± 5 K in the monolayer regime. The 2D crystalline structure is maintained up to 170 ± 5 K in the submonolayer and up to 225 ± 5 K in the monolayer regime. Hence, islands are thermally less stable than closed layers and the 2D glass structure is less stable than the 2D crystalline one.

On Ag(111), the melting temperature could only be determined for the 2D crystalline phase, where it was found to be 180 ± 10 K, both in the submonolayer and monolayer coverage regime. Because of the small amount of the 2D glass structure on the surface it was not possible to determine a defined melting point for the 2D glass structure, it definitely decays at lower temperatures than the 2D crystalline structure.

The thermal stability of the island is mainly determined by two parameters, by the surface diffusion barrier, i.e., the activation barrier for the motion of individual adsorbed species between two adjacent adsorption sides, and the interactions between adjacent adsorbates (adsorbate–adsorbate interactions). The fact that the IL adsorbates form islands at low temperatures is a clear proof for the existence of attractive adsorbate–adsorbate interactions between the adsorbed IL species. Furthermore it shows that the adsorbate–adsorbate interactions exceed the strength of the surface diffusion barrier, since otherwise the IL adsorbates would be trapped on their adsorption sites before they are able to undergo a 2D nucleation and growth process during cool-down to 100 K. Sufficient mobility of individual molecules is indicated also by the mobility at the island edges. Therefore, the temperature for 2D melting is dominated by the strength of the attractive adsorbate–adsorbate interactions. Interestingly, the trend in melting temperatures of the 2D crystalline phases on Ag(111) and Au(111) does not correlate with that of the adlayer density (see Table 1). While the 2D melting temperature on Ag(111) is only little higher than that of the 2D crystalline adlayer on Au(111) in the submonolayer regime, the density is comparable with that of the monolayer coverage adlayer on Au(111). This indicates that the adlayer stability is affected by the nature of the substrate, not only by purely distance (and thus density) dependent adsorbate–adsorbate interactions.

Since the structures in the 2D glass phase are similar for both substrates, we would expect the same melting temperature in both cases. It was not possible, however, to reliably determine the melting temperature of the 2D glass phase on Ag(111) (see above). For adsorption on Au(111), the lower melting temperature in the 2D glass phase compared to that in the 2D crystalline phase arises from the fact that the 2D glass phase is most likely a kinetically hindered structure and therefore not in thermodynamic equilibrium, which is less stable than the equilibrium phase. Interestingly, though the monolayer and the submonolayer coverage 2D glass adlayer have the same local density, the melting temperature of the latter is significantly lower. On the other hand, the melting temperature of the 2D glass phase at monolayer coverage and the 2D crystalline phase at submonolayer coverage, which also have similar densities, are essentially identical. In that case, the higher amount of defects in the former structure does not seem to play an important role.

## Conclusion

We have investigated substrate effects on the structure and structure formation, and thus on the substrate–adsorbate and adsorbate–adsorbate interactions, for the adsorption of [BMP][TFSA] by STM, by comparing their adsorption on the close-packed Au(111) and Ag(111) surfaces under UHV conditions in the temperature region between 100 K and 293 K. In combination also with previous data, these measurements lead to the following conclusions and adsorption characteristics:

1) Upon adsorption at room temperature, the [BMP][TFSA] adsorbates form a 2D gas/2D liquid phase with highly mobile adsorbed species on the surface. The integrity of the ions is maintained and both ions are in direct contact with the substrate surface. Interaction with the surface results in modifications of the electronic structure compared to that in condensed thicker layers. While XPS data exist only for adsorption on Au(111), we expect similar behavior also for adsorption on Ag(111).

2) Upon cooling the sample to 100 K, molecular motion in the adlayer is frozen and the adsorbates form islands/domains on the surface with 2D crystalline and 2D glass structures. In the submonolayer coverage regime, these coexist with (essentially) adsorbate free surface areas (2D gas). On Ag(111), the adsorbates form large islands consisting of a single domain of the 2D crystalline structure on terraces wider than ≈10 nm, while small terraces are (partly) covered with a 2D glass structure, and this phase dominates also in regions directly in front of ascending substrate steps. On Au(111), both structures are formed in small islands on the surface in the submonolayer regime. In the monolayer regime islands of the 2D crystalline phase are surrounded by the 2D glass phase. The 2D crystalline adlayer structure on Ag(111) is oriented along Ag surface lattice, with 3 different adlayer lattice orientations at angles of 120° to each other reflects the threefold symmetry of the Ag(111) substrate.

3) The 2D solid adlayer phases exhibit characteristic patterns consisting of round protrusions and longish protrusions in a ratio of 1:2. Based on comparison with results of previous DFT calculations [26], the round protrusion are identified as cations, with their ring lying flat on the surface and the butyl group pointing upwards, while the [TFSA] anions are represented by pairs of parallel longish protrusions. These mainly arise from the CF_3_ groups which are pointing upwards, while the anions bind to the surface with their O-atoms. Based on the similar structural characteristics in the STM images, we expect a similar adsorption geometry also for Au(111), where no DFT calculations exist.

4) Structure formation and adlayer structure/adlayer order are strongly affected by the reconstruction of the Au(111) substrate. Furthermore, they are also affected by the chemical nature of the substrate. The latter is reflected by the slightly different geometry (and IL adsorbate density) of the adlayer unit cell on the two surfaces, while the general appearance of the adlayer structure is identical on both substrate surfaces. The comparable density achieved on Au(111) in the monolayer coverage regime points to similar size substrate–adsorbate interactions on both surfaces. The influence of the reconstruction of the Au(111) surface is indicated in several ways: In addition to steps, the elbows of the Au(111) reconstruction act as preferential nucleation sites, starting island growth at these sites. Furthermore, they tend to induce narrow stripes of 2D glass phase in the adlayer when overgrown by an 2D crystalline adsorbate island/domain. The orientation of the 2D crystalline structure is also influenced by the Au(111) reconstruction pattern, it prefers to be oriented with the longer side of its unit cell along the Au(111) dislocation lines. Therefore domain boundaries of the adlayer structure often coincide with the connection line of adjacent elbows, where the Au(111) reconstruction pattern bends.

5) The (2D) melting temperature of the 2D solid phases is affected by substrate effects, by the adlayer coverage and by the order in the adlayer/domain. The melting temperature is significantly higher for the 2D crystalline phase on Au(111) than for the 2D glass phase, and it is higher in the (more closely packed) adlayer in the monolayer coverage regime than in the submonolayer coverage regime on the same substrate. For adsorption on Ag(111), where the density of the 2D crystalline phase does not depend on the overall coverage and where the size of the 2D crystalline islands is generally very large, we found no effects of the overall IL adsorbate coverage. The 2D melting temperature on Ag(111) resembles that on Au(111) in the submonolayer coverage regime, despite of the significantly lower density in the latter case. On the other hand, despite of similar densities on Ag(111) and Au(111) in the monolayer coverage regime (2D crystalline phase), the melting is significantly higher in the latter case, indicative of stronger (effective) adsorbate–adsorbate interactions on Au(111) than on Ag(111).

## Experimental

The measurements were performed in an UHV system with a base pressure of <4 × 10^−10^ mbar, equipped with an Aarhus type STM (SPECS; Aarhus STM 150), which allows measurements in the temperature range between 90 and 400 K, and standard facilities for surface preparation and surface characterization.

The Au(111) and Ag(111) samples were purchased from Mateck GmbH and cleaned by repeated sputtering with Ar^+^ (0.5 keV, 4 μA, 30 min) and heating to 770 K for 30 min, until atomically flat surfaces with mean terrace sizes of >100 nm were obtained (checked by STM). The Au(111) surface exhibited the typical reconstruction pattern with its characteristic regular zig-zag pattern [27]. Between two measurements, only a single cleaning cycle was sufficient to obtain a clean surface again.

The ionic liquid [BMP][TFSA] was purchased from Merck in ultrapure quality. It was mounted in a quartz crucible in a Knudsen effusion cell (Ventiotec, OVD-3) in the UHV chamber. It was degassed for more than one week in UHV at room temperature, followed by several hours degassing at 360 K. The crucible itself was also baked at 870 K in UHV before filling it with the IL. Prior to the experiments, the evaporation behaviour of [BMP][TFSA] was tested with a quartz micro balance. Based on these preliminary measurements, an evaporation temperature of 375 K was used in the experiments, which resulted in a pressure of 5 × 10^−10^ mbar. At this flow, a deposition time of 3 min resulted in a coverage of ca. 1 ML, as verified by STM. The cleanliness of the IL vapour was tested with a quadrupole mass spectrometer.

One monolayer is defined as one closed layer of ions in direct contact to the surface. In other publications [19,21], one closed layer of IL was defined as a layer of IL molecules with the cation and anion on top of each other, which gives 50% smaller values compared to our definition. These values were corrected to fit our definition in the present discussion.
